# Five New Species of Wood-Decaying Brown-Rot Fungi within Postiaceae (Polyporales, Basidiomycota) from Xinjiang, Northwest China

**DOI:** 10.3390/jof10090655

**Published:** 2024-09-17

**Authors:** Tai-Min Xu, Dong-Mei Wu, Neng Gao, Long Zeng, Yi-Hua Xu, Xiang-Ping Fan, Yi-Fei Sun, Bao-Kai Cui

**Affiliations:** 1 State Key Laboratory of Efficient Production of Forest Resources, School of Ecology and Nature Conservation, Beijing Forestry University, Beijing 100083, China; fungitaiminx@163.com (T.-M.X.); zenglong123123@163.com (L.Z.); yihuaxu0605@163.com (Y.-H.X.); fxp1343020708@163.com (X.-P.F.); yifeisun202209@bjfu.edu.cn (Y.-F.S.); 2 Xinjiang Production and Construction Group Key Laboratory of Crop Germplasm Enhancement and Gene Resources Utilization, Biotechnology Research Institute, Xinjiang Academy of Agricultural and Reclamation Sciences, Shihezi 832000, China; wdm0999123@sina.com (D.-M.W.); gaoneng520@163.com (N.G.)

**Keywords:** macrofungi, multigene, phylogeny, taxonomy, wood-decaying fungi

## Abstract

Brown-rot fungi are an important group of wood-decaying fungi, but there has been limited research on the species diversity of brown-rot fungi in Xinjiang, China. During an investigation of brown-rot fungi in Xinjiang, from July 2018 to July 2023, five new species belonging to the family Postiaceae were discovered based on morphological and molecular evidence. *Amaropostia altaiensis* is characterized by a conchate pileus, circular pores (5–8 per mm), and growing on *Populus*. *Amaropostia tianshanensis* is characterized by a flabelliform-to-conchate pileus, angular pores (5–6 per mm), and growing on *Picfea*. *Cyanosporus latisporus* is characterized by a hirsute and dark greyish blue pileal surface with fresh, larger pores (3–6 per mm) and broad basidiospores (4.3–5.9 × 1.4–2 µm). *Cyanosporus tianshanensis* is characterized by a smooth and white-to-cream pileal surface with fresh, smaller pores (6–9 per mm). *Osteina altaiensis* is characterized by a light mouse-grey-to-honey-yellow pileal surface, smaller pores (4–6 per mm), and slightly wide basidiospores (5–6 × 1.7–2.2 µm). Each of these five new species form independent lineages in phylogenetic analyses based on the seven gene loci (ITS + nLSU + nSSU + mtSSU + TEF1 + RPB1 + RPB2). This research enriches the diversity of brown-rot fungi species, while also demonstrating the substantial discovery potential and research value of brown-rot fungi in Xinjiang.

## 1. Introduction

Brown rot fungi are one of the important causes of wood degradation in forest ecosystems. They primarily decompose cellulose and hemicellulose in wood, leading to darkening, wrinkling, and characteristic block-like cracks, while their ability to degrade lignin is relatively weak. Gymnosperms have a higher proportion of cellulose, and the environmental conditions in temperate coniferous forests may better support the growth of brown-rot fungi, making them more prevalent in these forests [1,2,3]. Additionally, some species are edible and medicinal fungi, which have important economic value. In recent years, species diversity of the brown-rot fungi in China have been systematically investigated, and many species have been described [4,5,6,7,8,9,10,11,12,13,14,15]. Among these studies, several new species of the brown-rot fungi were described from Xinjiang, such as *Fomitopsis tianshanensis* B.K. Cui & Shun Liu, *Laetiporus xinjiangensis* J. Song, Y.C. Dai & B.K. Cui, and *Rhodonia tianshanensis* Yuan Yuan & L.L. Shen [8,14,16].

Xinjiang is located in the northwest of China. Its climate falls into the typical temperate continental climate category, characterized by large differences in daily and yearly temperatures, low annual precipitation, and extremely uneven distribution [17,18,19]. Due to the arid desert climate, the forest coverage in Xinjiang is low and unevenly distributed, typically found in mountainous areas with more precipitation, river valleys with more water, and areas with abundant groundwater [20]. The main tree species in Xinjiang are *Picea*, *Larix*, *Abies*, *Betula*, *Populus*, and *Salix* [20]. The unique environmental conditions and vegetation composition of Xinjiang have nurtured many large-scale endemic fungi. In recent years, with the continuous exploration of the Xinjiang region, an increasing number of fungal species have been discovered, greatly enhancing the diversity of macrofungi in Xinjiang. To date, Xinjiang has recorded over 600 species of macrofungi, with 247 species of polypores [21,22,23,24,25].

During further investigations of brown-rot fungi in Xinjiang, five new species of the brown-rot fungi were discovered, belonging to *Amaropostia* B.K. Cui, L.L. Shen & Y.C. Dai, *Cyanosporus* McGinty, and *Osteina* Donk in Postiaceae B.K. Cui, Shun Liu & Y.C. Dai. Postiaceae was established to integrate the *Postia* genus and its related taxa, and all species in this family can cause brown-rot. Currently, there are a total of 17 genera and 97 species, including 14 genera and 67 species found in China [7,10]. The genus *Amaropostia* was established by Shen et al. [13]; the genus is characterized by soft corky basidiocarps when fresh, woody hard when dry; a bitter taste; a white- or cream-to-buff pileal surface; and cylindrical basidiospores [13]. The genus *Cyanosporus* was established by McGinty [26] based on morphological analyses to accommodate to *Cyanosporus caesius* (Schrad.) McGinty. The genus is characterized by pileate- or resupinate-to-effused–reflexed basidiocarps; a white- or cream-to-greyish-brown pileal surface, usually with a blue tint; a white-to-cream pore surface, frequently bluish; and round-to-angular pores [11]. To date, a total of 35 species of *Cyanosporus* are accepted in the world; China is the main distribution area of *Cyanosporus*, with 21 recorded species. The genus *Osteina* was established by Donk [27]; the genus is characterized by sessile to distinctly stipitate basidiocarps, which are bone hard when dry, a monomitic hyphal system with clamped generative hyphae, and hyaline and thin-walled basidiospores without any reactions in Melzer’s and Cotton Blue reagents [5]. Phylogenetic analyses were carried out based on multi-gene sequences (ITS + nLSU + nSSU + mtSSU + TEF1 + RPB1 + RPB2), which indicated that the new species belong to *Amaropostia* B.K. Cui, L.L. Shen & Y.C. Dai, *Cyanosporus* McGinty, and *Osteina* Donk in Postiaceae. The new species were described based on the combination of morphological and phylogenetic analyses.

## 2. Materials and Methods

### 2.1. Morphological Studies

The specimens examined in this research are housed in the herbarium at the Institute of Microbiology, Beijing Forestry University, China (BJFC). Macroscopic features were documented from field observations and subsequent laboratory assessments. The microscopic techniques employed in this study adhere to the protocols established by Cui et al. [28] and Liu et al. [7]. Examination of the sections was conducted using a Nikon E80i microscope with phase contrast illumination, capable of magnifications up to 1000×, manufactured by the Nikon Corporation in Tokyo, Japan. Line illustrations were created utilizing a drawing tube attachment. The microscopic characteristics, measurements, and illustrations were derived from slide preparations of either dried or fresh material, stained using Cotton Blue and Melzer’s reagent, following the staining procedures detailed by Sun et al. [29]. To illustrate the range of basidiospore sizes, the extreme 5% of measurements were omitted from the reported range and are indicated in parentheses. The text employs the following abbreviations: IKI for Melzer’s reagent, IKI− indicating the absence of dextrinoid or amyloid properties, KOH for a 5% solution of potassium hydroxide, CB for Cotton Blue, CB+ for the cyanophilous reaction, CB− for the acyanophilous reaction, L for the mean length of spores (calculated as the arithmetic average), W for the mean width of spores (calculated as the arithmetic average), Q for the variability in the L/W ratio among the samples, and n for the total number of spores measured across multiple samples. The color terminology followed Petersen [30].

### 2.2. DNA Extraction, PCR, and Sequencing

Utilizing a CTAB-based rapid plant genome extraction kit (DN14) from Aidlab Biotechnologies in Beijing, China, we isolated the total genomic DNA from dried specimens. Subsequent polymerase chain reaction (PCR) was conducted, adhering to the kit manufacturer’s guidelines with adjustments as per the methodologies of Sun et al. [31] and Ji et al. [32]. The internal transcribed spacer (ITS) regions were amplified with primer pairs ITS5 and ITS4 [33]. The large subunit nuclear ribosomal RNA gene (nLSU) regions were amplified with primer pairs LR0R and LR7 [34]. The small subunit mitochondrial rRNA gene sequence (mtSSU) regions were amplified with primer pairs MS1 and MS2 [33]. The small subunit nuclear ribosomal RNA gene (nSSU) regions were amplified with primer pairs NS1 and NS4 [33]. The RNA polymerase II largest subunit (RPB1) was amplified with primer pairs RPB1-Af and RPB1-Cr [35]. The second largest subunit (RPB2) was amplified with primer pairs bRPB2-6F and bRPB2-7R [36]. The translation elongation factor 1-α gene (TEF1) was amplified with primer pairs EF1-983F and EF1-1567R [37]. The PCR procedures for seven gene fragments were followed by Liu et al. [10] and Sun et al. [33] in the phylogenetic analyses. The PCR products were subsequently purified and sequenced at the Beijing Genomics Institute (BGI), employing the original primers used in amplification. The resulting novel sequences have been deposited in the GenBank database, with details provided in the referenced Table 1.

### 2.3. Phylogenetic Analyses

The sequences generated in this study and retrieved from GenBank were combined with ITS, nLSU, mtSSU nuSSU, RPB1, RPB2, and TEF1. The species *Antrodia serpens* (Fr.) P. Karst. and *Fomitopsis betulina* (Bull.) B.K. Cui, M.L. Han & Y.C. Dai were used as outgroups [7]. All sequences of ITS, nLSU, mtSSU nuSSU, RPB1, RPB2, and TEF1 were aligned in MAFFT v. 7 (https://mafft.cbrc.jp/alignment/server/index.html, accessed on 22 July 2024) and manually adjusted in BioEdit [44,45]. Alignments were spliced in Mesquite v. 3.2. [46]. A phylogenetic analysis of the five new species of Postiaceae was conducted using a combination of methods, including maximum likelihood (ML), maximum parsimony (MP), and Bayesian inference (BI), all based on a dataset that integrates ITS, nLSU, mtSSU, nSSU, RPB1, RPB2, and TEF1 sequences. The phylogenetic analyses used in this study followed the approach of Ji et al. and Sun et al. [31,32].

The procedure of maximum likelihood (ML) estimation was executed using RAxML-HPC252, accessed via the CIPRES Science Gateway portal at www.phylo.org, encompassing a series of 100 ML inquiries. All model parameters were estimated by the program. From the array of maximum likelihood trees generated, the one deemed to be the most optimal was preserved. Additionally, the bootstrap values for maximum likelihood (ML-BS) were ascertained employing a quick bootstrapping approach, conducting the process 1000 times to obtain a reliable evaluation.

The maximum parsimony (MP) technique was executed in PAUP* 4.0b10 [47]. The phylogenetic analysis parameters were configured in accordance with the research methodologies proposed by Ji et al. [32]. Each character was given the same importance in the analysis, with gaps in the sequence being considered absent data. The tree constructions were deduced employing a heuristic search method, which included Tree Bisection and Reconnection (TBR) swaps and initiated with an input of 1000 randomly chosen sequences. The analysis parameters were set to a maximum of 5000 trees, collapsing branches with zero length and preserving all resulting parsimonious trees for examination. The robustness of the clades was determined by a bootstrap analysis with 1000 replications [48]. For each maximum parsimonious tree (MPT) that was produced, a set of descriptive statistical measures was computed, including tree length (TL), consistency index (CI), retention index (RI), the rescaled consistency index (RC), and homoplasy index (HI). These indices provided a quantitative assessment of the trees’ phylogenetic structure and support [49].

The Bayesian inference (BI) method was utilized with the software MrBayes, version 3.2.6 [50]. This analysis consisted of two separate runs, each involving four simultaneous chains. The chains were run for a total of 5,000,000 generations, with samples taken from the posterior distribution every 1000 generations. This sampling frequency was employed to ensure that the potential scale reduction factors (PSRFs) approached 1.0 for all parameters, which is indicative of convergence among the chains. To account for initial instability in the chains, the initial 25% of the sampled trees were excluded from further analysis. Subsequently, the remaining trees were employed to determine the Bayesian posterior probabilities (BPPs) for each distinct group. Ultimately, a consensus tree was constructed by aggregating all the remaining trees, adhering to the principle of majority rule.

Branches with bootstrap support exceeding or equaling 50% in both maximum parsimony (MP) and maximum likelihood (ML) analyses, as well as Bayesian posterior probabilities (BPPs) of 0.90 or above, were regarded as robustly supported. Phylogenetic trees were visualized using FigTree v1.4.2.

## 3. Results

### 3.1. Molecular Phylogeny

**Figure 1:**
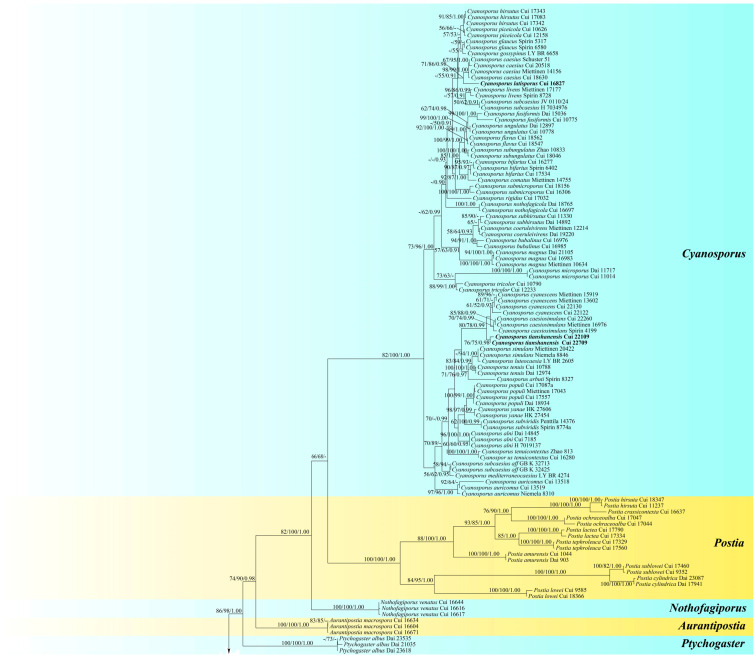

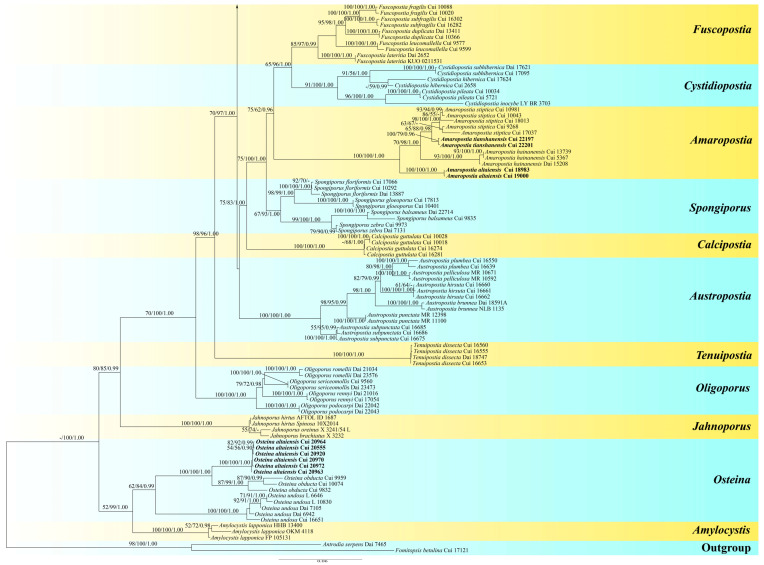
Maximum likelihood (ML) tree of Postiaceae based on the combined ITS+nLSU+mtSSU+nSSU+RPB1+RPB2+TEF1 dataset. Branches are labelled with maximum parsimony/maximum likelihood bootstrap values higher than 50% and Bayesian posterior probability values greater than 0.90. New species are indicated in bold.

The phylogenetic analyses of the new species within Postiaceae were conducted based on a combined ITS + nLSU + mtSSU + nSSU + RPB1 + RPB2 + TEF1 dataset. This dataset comprised a total of 196 ITS, 164 nLSU, 145 mtSSU, 149 nSSU, 116 RPB2, 101 RPB1, and 142 TEF1 gene sequences derived from 198 fungal specimens, encompassing 88 species. The compiled dataset consisted of 6562 characters, with 3921 being constant across all sequences, 427 being variable yet not contributing phylogenetic information in terms of parsimony, and 2204 being positions that were informative for parsimony, providing the necessary variation to infer evolutionary relationships. MP analysis resulted in ten trees of equal parsimony (TL = 12702, CI = 0.3415, RI = 0.8227, RC = 0.2810, HI = 0.6585). In the Bayesian inference analysis, the most suitable model selected was GTR + I + G. The topological structure obtained from BI was congruent with that from MP and ML analysis. BI exhibited an average standard deviation of split frequencies at 0.008999; only the ML topology is shown in Figure 1.

In the phylogenetic analyses (Figure 1), a total of 16 genera of Postiaceae were included, and their names were labeled on the right side of the tree. The phylogenetic results indicated that the five new species collected from Xinjiang are distinct from other known species. *Amaropostia altaiensis* and *A. tianshanensis* stably clustered on branches of *Amaropostia*; Among them, *A. tianshanensis* was more closely clustered with *A. stiptica* (Pers.) B.K. Cui, L.L. Shen & Y.C. Dai, with nine different nucleobases in its ITS sequence, and the similarity was 98.39% according to nucleotide BLAST. *Amaropostia tianshanensis* was more distantly clustered with *A. hainanensis* B.K. Cui, L.L. Shen & Y.C. Dai, with 54 different nucleobases in its ITS sequence, and the similarity was 90.65% according to nucleotide BLAST. Meanwhile, *A. altaiensis* was more distantly clustered from the other two known species; it had 74 different nucleobases in its ITS sequence with *A. hainanensis*, and the similarity was 92.29% according to nucleotide BLAST; it had 43 different nucleobases with *A. stiptica*, and the similarity was 87.14% according to nucleotide BLAST.

*Cyanosporus latisporus* and *C. tianshanensis* stably clustered on branches of *Cyanosporus*. Among them, *C. latisporus* clustered with *C. caesius* on a single branch, with 14 different nucleobases in their ITS base sequences, and the similarity was 97.69%, as determined by nucleotide BLAST. *C. tianshanensis* was grouped with *C. caesiosimulans* (G.F. Atk.) B.K. Cui & Shun Liu and *C. cyanescens* (Miettinen) B.K. Cui & Shun Liu on a branch, with 17 different nucleobases in the ITS base sequence between *C. tianshanensis* and *C. caesiosimulans*, and the similarity was 97.4% by nucleotide BLAST; between *C. tianshanensis* and *C. cyanescens*, there were also 17 different nucleobases in the ITS base sequence, and the similarity was 97.5% by nucleotide BLAST.

In addition, *Osteina altaiensis* stably clustered on branches of *Osteina*. Among them, *O. altaiensis* was more closely related to *O. obducta* (Berk.) Donk, with 36 different nucleobases in their ITS base sequences, and the similarity was 93.17% as determined by nucleotide BLAST. In comparison with *O. undosa* (Peck) Zmitr., there were 100 different nucleobases in the ITS base sequence, and the similarity was 83.92% by nucleotide BLAST.

### 3.2. Taxonomy

**Figure 2:**
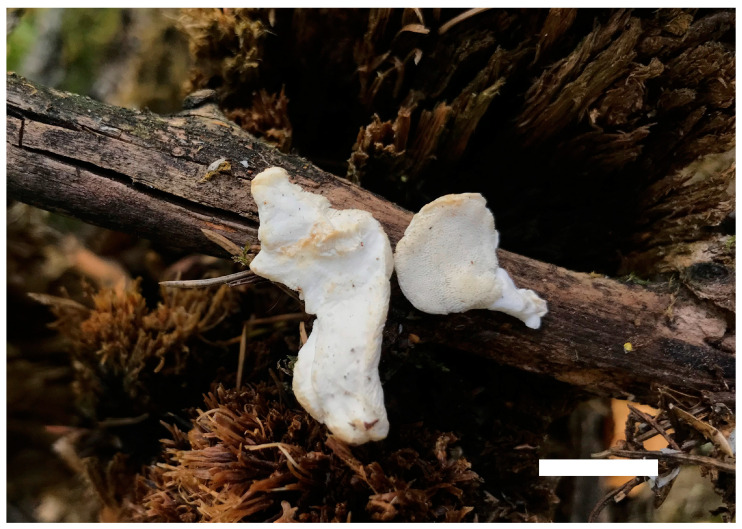
Basidiomata of *Amaropostia altaiensis* (Cui 19000). Scale bar: 1 cm.

**Figure 3:**
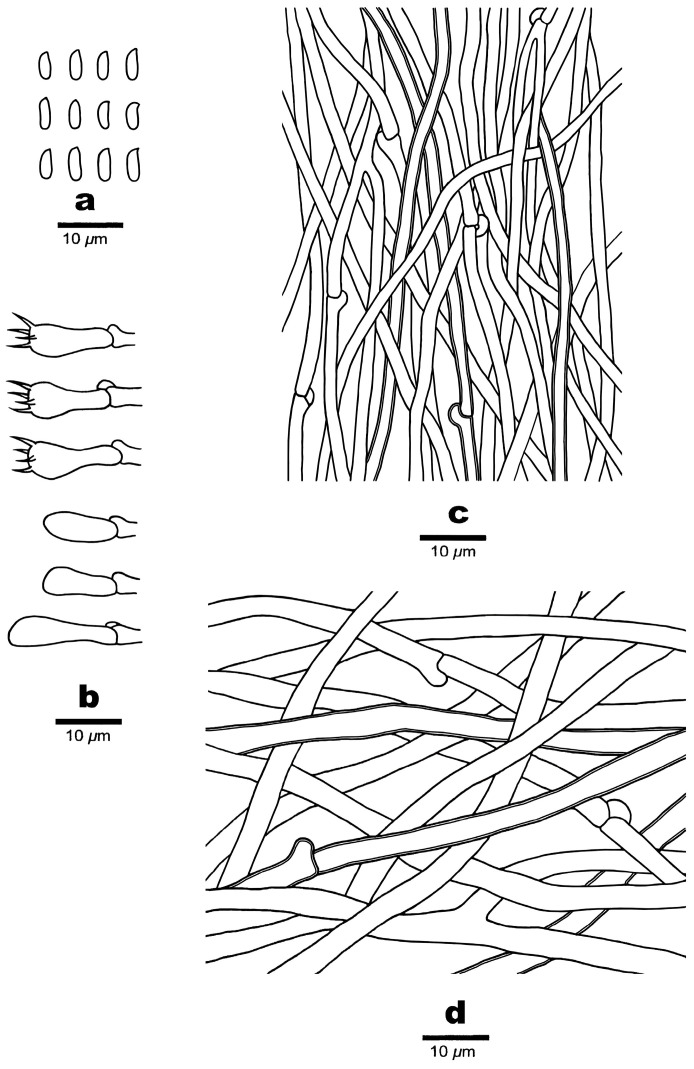
Microscopic structures of *Amaropostia altaiensis* (Cui 19000). (**a**) Basidiospores; (**b**) basidia and basidioles; (**c**) hyphae from trama; and (**d**) hyphae from context.

***Amaropostia altaiensis*** B.K. Cui, Y.F. Sun & T.M. Xu, sp. nov. Figure 1, Figure 2 and Figure 3.

MycoBank: 855779

Diagnosis: *Amaropostia altaiensis* differs from other species in the genus by its conchate pileus, slightly broad basidiospores, larger pores, and host *Populus* sp.

Holotype: China, Xinjiang Uyghur Autonomous Region, Burqin County, Altai Mountains, Hemu, alt. 1057–1150 m, on a fallen branch of *Populus* sp., 19 September 2021, Cui 19000 (BJFC).

Etymology: *altaiensis* (Lat.): refers to the species’ occurrence in the Altai Mountains.

Basidiomata: annual, sessile, or has an indistinct stipe in base; solitary; soft when fresh, becoming corky to woody hard when dry; has a distinctly bitter taste; and its pileus is flabelliform to spathulate, projecting up to 18 mm, 22 mm wide, and 7 mm thick at base. Pileal surface is white when in a fresh state, changing from cream (4A2/3) to buff (4A4) when dry; margins are slightly obtuse. Pore surface is white when fresh, changing from cream (4A2/3) to clay-buff (6D4) when dry, with sterile margins narrow to almost lacking; pores are angular, 5–8 per mm; and dissepiments are thin and entire. Context is white to cream (4A2/3), woody hard, and up to 0.7 cm thick. Tubes are white to cream (4A2/3), corky, and up to 0.3 cm long.

Hyphal structure: Hyphal system is monomitic; generative hyphae are clamped, IKI−, CB−; and tissues are unchanged in KOH.

Context: Generative hyphae hyaline, thin-walled to slightly thick-walled, occasionally branched, interwoven, and 3–4 μm in diam.

Tubes: Generative hyphae hyaline, thin-walled to slightly thick-walled, occasionally branched, interwoven, and 2–3 µm diam. Cystidia or cystidioles are absent. Basidia are clavate, with four spores at the apex, clamped at base, and 13–19 × 5–6 µm; basidioles are in a shape similar to basidia but slightly smaller.

Spores: Basidiospores are cylindrical, slightly curved, hyaline, thin-walled, smooth, usually tapering at apiculus, IKI−, CB−, 4–5.5 × 1.6–2.2 μm, L = 4.85 μm, W = 1.93 μm, and Q = 2.48–2.52 (n = 60/2).

Type of rot: Brown rot.

Additional specimens (paratype) examined: China, Xinjiang Uyghur Autonomous Region, Burqin County, Altai Mountains, Hemu, alt. 1057–1150 m, on fallen trunk of *Populus* sp., 19 September 2021, Cui 18983 (BJFC).

**Figure 4:**
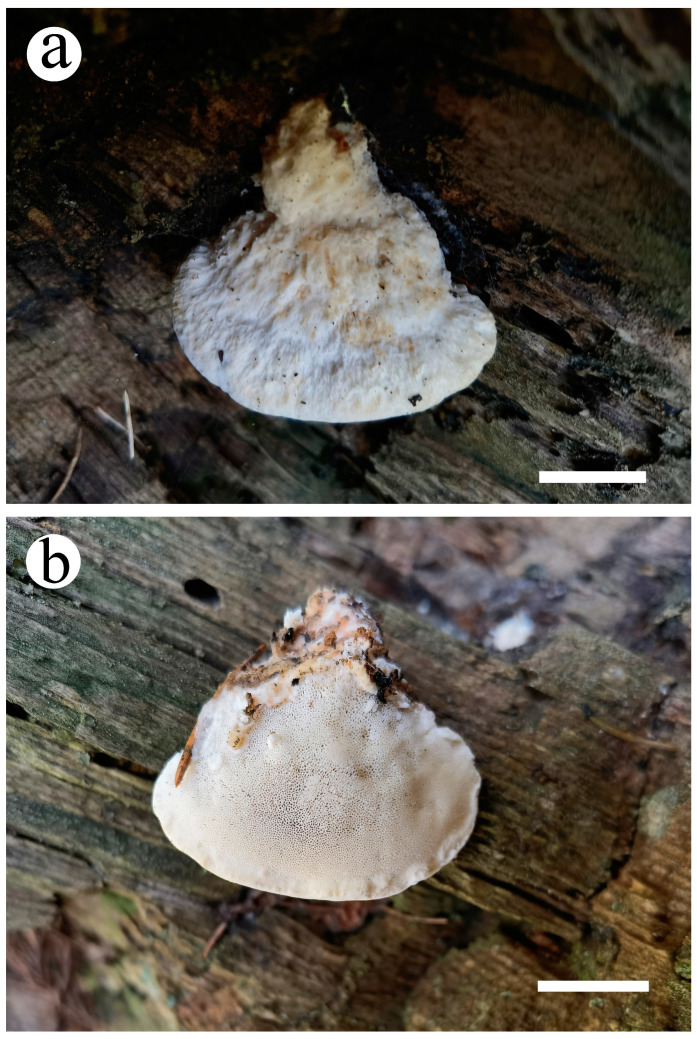
Basidioma of *Amaropostia tianshanensis* (Cui 22201). (**a**) Upper surface of the basidiomata; (**b**) lower surface of the basidiomata. Scale bar = 1 cm (**a**,**b**).

**Figure 5:**
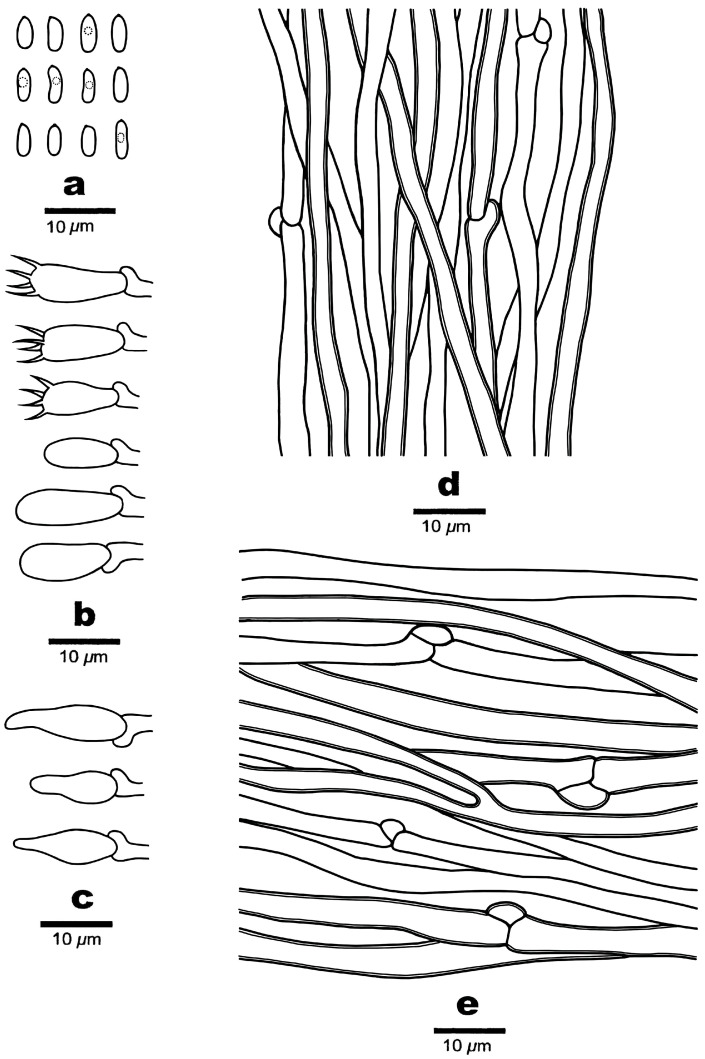
Microscopic structures of *Amaropostia tianshanensis* (Cui 22201). (**a**) Basidiospores; (**b**) basidia and basidioles; (**c**) cystidioles; (**d**) hyphae from trama; and (**e**) Hyphae from context.

***Amaropostia tianshanensis*** B.K. Cui, Y.F. Sun & T.M. Xu, sp. nov. Figure 1, Figure 4 and Figure 5.

MycoBank: 855780

Diagnosis: *Amaropostia tianshanensis* differs from other species in the genus by a flabelliform-to-conchate pileus, larger pores, and slightly broad basidiospores.

Holotype: China. Xinjiang Uyghur Autonomous Region, Tekes County, Tianshan Mountains, Kosang Cave National Forest Park, alt. 1977 m, on fallen trunk of *Picea schrenkiana* Fisch. & C. A. Mey., 22 July 2023, Cui 22201 (BJFC).

Etymology: *tianshanensis* (Lat.): refers to the species occurrence in Tianshan Mountains.

Basidiomata are annual, sessile, solitary, soft when fresh, becoming corky to woody hard upon drying, and have a distinctly bitter taste; pileus is flabelliform to conchate, projecting up to 6.5 cm, 3.5 cm wide, and 1.2 cm thick at base. Pileal surface white to cream (4A2/3) in its fresh state, changing from cream (4A2/3) to buff-yellow (4A4) upon drying; margin obtuse. Pore surface is white to cream (4A2/3) when fresh, with a slightly buff-yellow (4A4) at the base, changing from buff (4A4) to honey yellow (4/5B4) when dry, with sterile margins narrow to almost lacking; pores are circular to irregular, 5–6 per mm; and dissepiments are thin and entire. Context is white, woody hard, and up to 0.8 cm thick. Tubes are white, corky, and up to 0.6 cm long.

Hyphal structure: Hyphal system is monomitic; generative hyphae are clamped, IKI−, CB−; and tissues are unchanged in KOH.

Context: Generative hyphae hyaline, thin-walled to slightly thick-walled, occasionally branched, interwoven, and 2.5–4.2 μm diam.

Tubes: Generative hyphae hyaline, thin-walled to slightly thick-walled, occasionally branched, interwoven, and 2–3.8 µm diam. Cystidia are absent; fusoid cystidioles are present, hyaline, thin-walled, and 10.8–16.4 × 2.4–4 μm. Basidia are clavate, four spores at the apex, clamped at base, and 11–14.2 × 3.7–5.2 µm; basidioles are in a shape similar to basidia but slightly smaller.

Spores: Basidiospores are cylindrical, hyaline, thin-walled, smooth, IKI−, CB+, 3.9–5.8 × 1.5–2.3(2.6) μm, L = 4.58 μm, W = 1.81 μm, and Q = 2.45–2.68 (n = 90/3).

Type of rot: Brown rot.

Additional specimens (paratypes) examined: China. Xinjiang Uyghur Autonomous Region, Tekes County, Tianshan Mountains, Kosang Cave National Forest Park, alt. 2000 m, on fallen trunk of *Picea schrenkiana*, 22 July 2023, Cui 22197 (BJFC).

**Figure 6:**
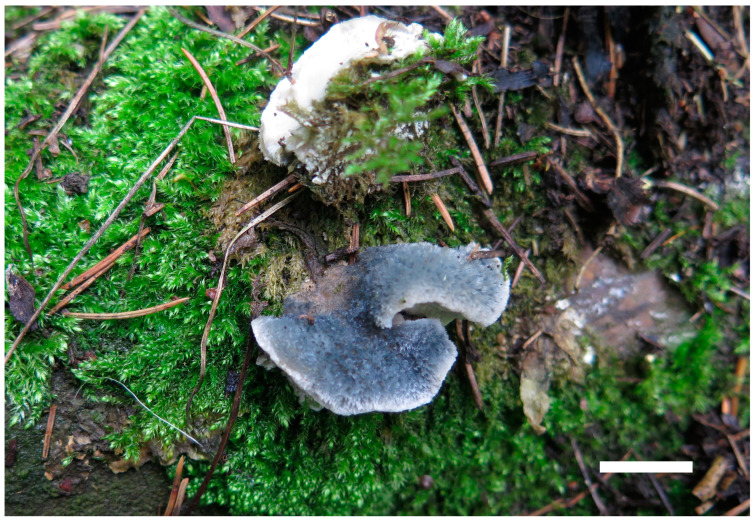
Basidiomata of *Cyanosporus latisporus* (Cui 16827). Scale bar: 1 cm.

**Figure 7:**
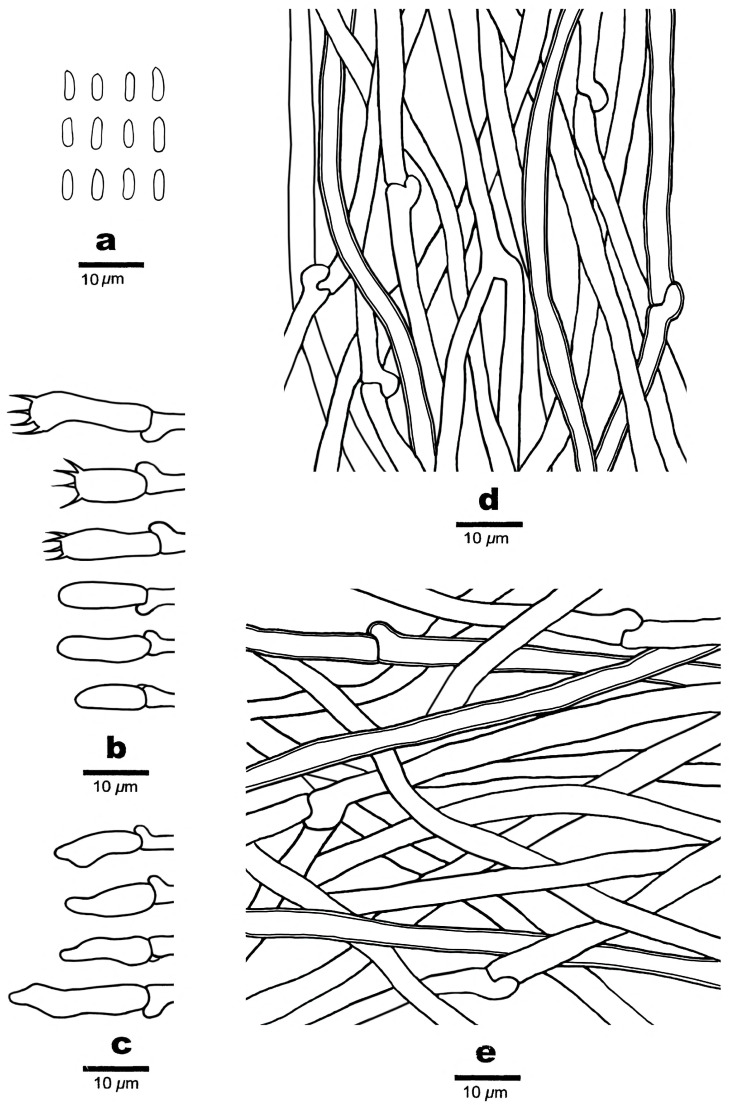
Microscopic structures of *Cyanosporus latisporus* (Cui 16827). (**a**) Basidiospores; (**b**) basidia and basidioles; (**c**) cystidioles; (**d**) hyphae from trama; and (**e**) Hyphae from context.

***Cyanosporus latisporus*** B.K. Cui, Y.F. Sun & T.M. Xu, sp. nov. Figure 1, Figure 6 and Figure 7.

MycoBank: 855781

Diagnosis: *Cyanosporus latisporus* differs from other species in the genus by a dark greyish blue pileal surface, larger pores, and broad basidiospores.

Holotype: China. Xinjiang Uyghur Autonomous Region, Urumqi County, Nanshan Scenic Spot, Miaoergou, alt. 2060 m, on fallen trunk of *Picea schrenkiana*, 5 July 2018, Cui 16827 (BJFC).

Etymology: *latisporus* (Lat.): refers to the broad basidiospores.

Basidiomata are annual, pileate, solitary or imbricate, soft, corky, odorless, and tasteless when fresh, changing from corky to fragile and light in weight when drying. Pileus is semicircular to conchate, projecting up to 2 cm, 3 cm wide, and 1.6 cm thick at base. Pileal surface is hirsute, dark greyish blue (19D/E5) when fresh, becoming ash-grey (19C2) when dry; margin is acute. Pore surface is white when fresh, changing from cream (4A2/3) to buff (6D4) upon dry; sterile margins are narrow to almost lacking; pores are irregular, 3–6 per mm; dissepiments are thin, entire to slightly lacerate. Context is white, soft corky, and up to 1 cm thick. Tubes are white to cream (4A2/3), fragile, and up to 3 mm long.

Hyphal structure: Hyphal system is monomitic; generative hyphae are clamped, IKI−, CB−; and hyphae are unchanged in KOH.

Context: Generative hyphae hyaline, thin-walled to slightly thick-walled, occasionally branched, loosely interwoven, and 2.5–4 μm in diam.

Tubes: Generative hyphae hyaline, thin-walled to slightly thick-walled, occasionally branched, interwoven, and 2.5–4 μm in diam. Cystidia are absent; cystidioles are present, fusoid, thin-walled, and 13–20 × 3.5–4.5 μm. Basidia are clavate, with four spores at the apex, and with a basal clamp connection, 10.5–18.3 × 3.5–5.1 μm; basidioles in shape are similar to basidia but slightly smaller.

Spores: Basidiospores are allantoid to cylindrical, hyaline, thin-walled to slightly thick-walled, smooth, IKI−, CB+, 4.3–5.9 × 1.4–2(2.3) μm, L = 4.79 μm, W = 1.74 μm, and Q = 2.75 (n = 30/1).

Type of rot: Brown rot.

**Figure 8:**
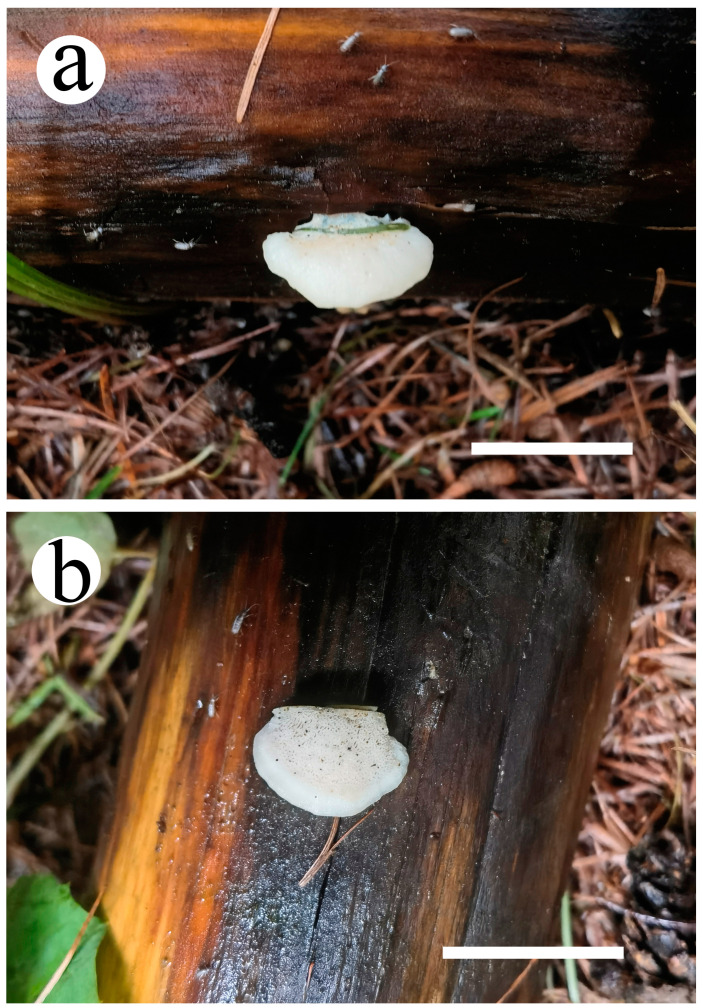
Basidioma of *Cyanosporus tianshanensis* (Cui 22709). (**a**) Upper surface of the basidiomata; (**b**) lower surface of the basidiomata. Scale bar = 1.0 cm (**a**,**b**).

**Figure 9:**
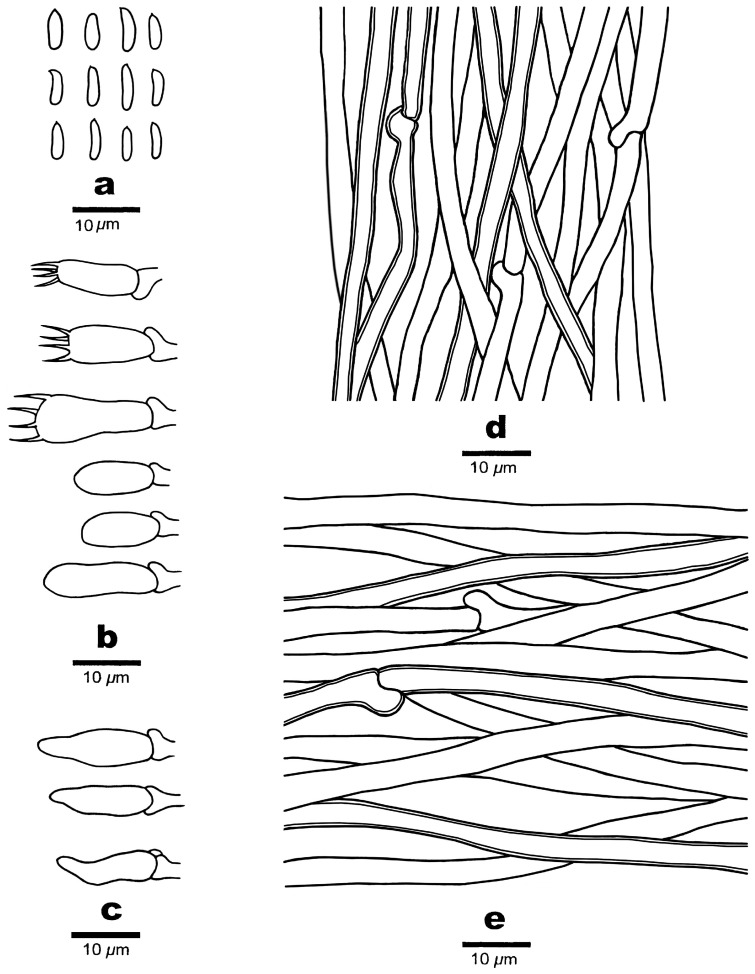
Microscopic structures of *Cyanosporus tianshanensis* (Cui 22709). (**a**) Basidiospores; (**b**) basidia and basidioles; (**c**) cystidioles; (**d**) hyphae from trama; and (**e**) hyphae from context.

***Cyanosporus tianshanensis*** B.K. Cui, Y.F. Sun & T.M. Xu, sp. nov. Figure 1, Figure 8 and Figure 9.

MycoBank: 855782

Diagnosis: *Cyanosporus tianshanensis* differs from other species in the genus by a smooth pileal surface and smaller pores.

Holotype: China. Xinjiang Uyghur Autonomous Region, Wensu County, Tianshan Mountains, Tomur National Nature Reserve, Xiaokuzibayi area, alt. 2493 m, on fallen trunk of *Larix* sp., 27 July 2023, Cui 22709 (BJFC).

Etymology: *tianshanensis* (Lat.): refers to the species’ occurrence in Tianshan Mountains.

Basidiomata are annual, pileate, soft corky, solitary, odorless, and tasteless when fresh, changing from corky to fragile and light in weight when drying. Pileus is conchate or irregular, projecting up to 1.7 cm, 1.2 cm wide, and 0.6 cm thick at base. Pileal surface is smooth, white to cream (4A2/3), with a small amount of grey blue (20C5) at base when fresh, changing from buff (6D4) to pale mouse grey (7C2) when dry; margins are acute. Pore surface is cream (4A2/3) when fresh, changing to buff (6D4) when dry; sterile margins are white, up to 1.5 mm; pores are irregular, 6–9 per mm; and dissepiments are thin, entire to slightly lacerate. Context is white, soft corky, and up to 0.5 mm thick. Tubes are white to cream (4A2/3), fragile, and up to 1 mm long.

Hyphal structure: Hyphal system is monomitic; generative hyphae are clamped, IKI−, CB−; and hyphae are unchanged in KOH.

Context: Generative hyphae hyaline, thin-walled to slightly thick-walled, occasionally branched, loosely interwoven, and 2.6–4.2 μm in diam.

Tubes: Generative hyphae hyaline, thin-walled to slightly thick-walled, occasionally branched, interwoven, and 2–4 μm in diam. Cystidia are absent; cystidioles are present, fusoid, thin-walled, and 13–16.5 × 4–5.5 μm. Basidia are clavate, with four spores at the apex, and a basal clamp connection, 10.8–17.3 × 3.7–5.1 μm; basidioles are clavate and distinctly smaller than basidia.

Spores: Basidiospores are cylindrical, lightly curved, hyaline, thin-walled to slightly thick-walled, smooth, IKI−, CB+, 4.4–5.9 × 1.3–1.7 μm, L = 5.18 μm, W = 1.48 μm, and Q = 3.36–3.63 (n = 60/2).

Type of rot: Brown rot.

Additional specimens (paratype) examined: China. Xinjiang Uyghur Autonomous Region, Zhaosu County, Tianshan Mountains, Xiata National Forest Park, alt. 2370 m, on fallen trunk of *Picea schrenkiana*, 21 July 2023, Cui 22109 (BJFC).

**Figure 10:**
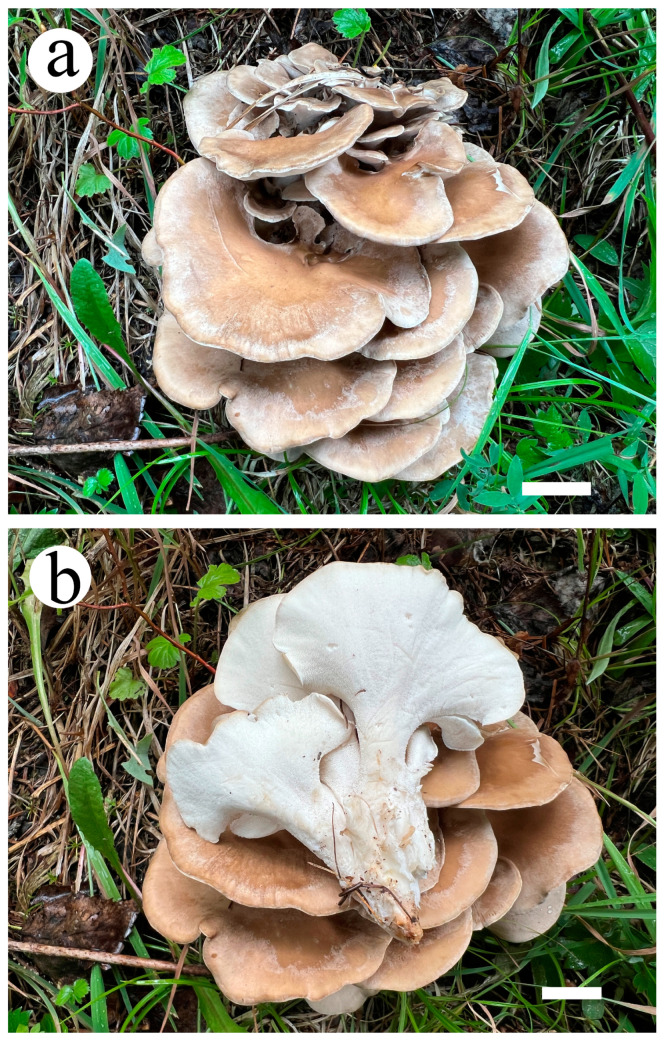
Basidiomata of *Osteina altaiensis* (Cui 20920). (**a**) Upper surface of the basidiomata; (**b**) lower surface of the basidiomata. Scale bar = 2 cm (**a**,**b**).

**Figure 11:**
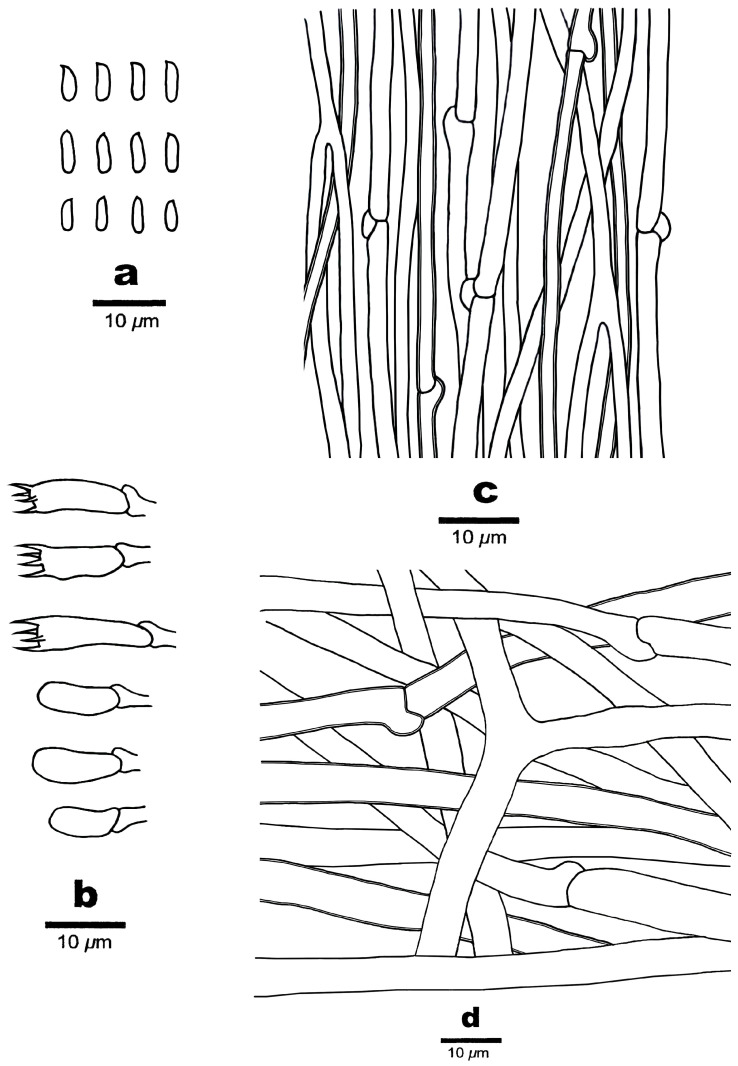
Microscopic structures of *Osteina altaiensis* (Cui 20920). (**a**) Basidiospores; (**b**) basidia and basidioles; (**c**) hyphae from trama; and (**d**) hyphae from context.

***Osteina altaiensis*** B.K. Cui, Y.F. Sun & T.M. Xu, sp. nov. Figure 1, Figure 10 and Figure 11.

MycoBank: 855783

Diagnosis: *Osteina altaiensis* differs from other species in the genus by a light mouse-grey-to-honey-yellow pileal surface, smaller pores, and slightly broad basidiospores.

Holotype: China, Xinjiang Uyghur Autonomous Region, Burqin County, Altai Mountains, Jiadengyu National Forest Park, alt. 1751–1861 m, on the root of *Larix* sp., 22 July 2022, Cui 20920.

Etymology: *altaiensis* (Lat.): refers to the species’ occurrence in Altai Mountains.

Basidiomata are annual, usually laterally stipitate, and branches multiple times, forming a cluster of imbricate pilei and flesh and without special odor or taste when fresh, becoming bone hard and having a slight pungent smell when dry. Pilei are semicircular or flabelliform, up to 15 cm long, 10 cm wide, and 3 cm thick; margins are acute, undulate, and curved down when dry. Pileal surface is light mouse grey (9F3) to honey yellow (4/5B4) when fresh; margins are usually paler than the center, smooth, and azonate, changing to hazel (6E8) and wrinkled after dry. Pore surfaces are white when fresh, becoming buff (6D4) when dry; pores are angular to irregular, 4–6 per mm; and dissepiments are thin and entire. The base is salmon pink when fresh, changing to cinnamon when dry. Context is white and flesh-white when fresh, azonate, changing from hard corky to rigid when dry, and up to 5 cm thick. Tubes are white when fresh, buff (6D4) upon drying, and up to 1.5 mm long.

Hyphal structure: Hyphal system is monomitic; generative hyphae are clamped, IKI−, CB−; and tissues are unchanged in KOH.

Context: Generative hyphae hyaline, thin-walled to slightly thick-walled, occasionally branched, interwoven, and 5–10 μm in diam.

Tubes: Generative hyphae hyaline, thin-walled to slightly thick-walled, occasionally branched, interwoven, and 2–3 μm in diam. Cystidia and cystidioles are absent. Basidia are clavate, with four spores at the apex, clamped at base, and 13–15 × 4–5.5 μm; basidioles in shape are similar to basidia but slightly smaller.

Spores: Basidiospores are cylindrical, slightly tapering at apiculus, hyaline, thin-walled, smooth, smooth, IKI−, CB−, 5–6 × 1.7–2.2 μm, L = 5.15 μm, W = 2.02 μm, and Q = 2.52–2.72 (n = 270/9).

Type of rot: Brown rot.

Additional specimens (paratypes) examined: China, Xinjiang Uyghur Autonomous Region, Burqin County, Altai Mountains, Jiadengyu National Forest Park, alt. 1751–1861 m, on root of *Picea* sp., 20 September 2021, Cui 20481A, Cui 20555, Cui 20601; on root of *Larix* sp., 20 September 2021, Cui 20955, Cui 20963, Cui 20964, Cui 20970, Cui 20972.

## 4. Discussion

According to the phylogenetic analyses based on the combined seven-gene dataset, *Amaropostia altaiensis* and *A. tianshanensis* were involved in *Amaropostia* with strong support (100% ML, 100% MP, 1.00 BPPs). *Amaropostia altaiensis* constitutes a unique branch in the phylogenetic tree, which is clearly distinguished from all other known species of the genus. Morphologically, *Amaropostia hainanensis* and *A. altaiensis* are similar, both having pileate basidiocarps and a white-to-buff pore surface, and grow on angiosperms; however, *A. hainanensis* differs in having angular and smaller pores (7–9 per mm), narrower spores (4–5.5 × 1.5–2 µm, Q = 2.59–2.73), and distribution in tropical areas [13]. *Amaropostia stiptica* resembles *A. altaiensis* in having similar pore sizes and a white-to-buff pore surface, but *A. stiptica* differs in having effused–reflexed-to-pileate basidiocarp, broader basidiospores (4–4.5 × 1.5–2 µm, Q = 2.19–2.38), and fusoid cystidioles [13,51]. *Amaropostia tianshanensis* and *A. altaiensis* contain similarity in being conchate basidiocarp, with similar sizes and shapes of pore and spore, but *A. tianshanensis* has angular pores and grows on gymnosperms. *Amaropostia tianshanensis* and *A. stiptica* may be confused morphologically, as they share similarities in basidiocarp color and size, pore size, and fusoid cystidioles, but *A. stiptica* has an effused–reflexed-to-pileate basidiocarp and broader basidiospores [13].

*Cyanosporus latisporus* and *C. tianshanensis* were involved in *Cyanosporus* with strong support (100% ML, 100% MP, 1.00 BPPs) in the phylogenetic analyses. *Cyanosporus caesius* and *C. latisporus* are closely related phylogenetically. Morphologically, they are also very similar, with bluish-greyish caps, hirsute pileal surface, and a white pore surface when fresh, with similar pore sizes, but *C. caesius* differs in having slightly lighter-colored basidiocarps and narrower basidiospores (4.1–5.3 × 1.3–1.7 µm, Q = 3.18–3.29, [40]). *Cyanosporus tianshanensis* is closely related to *C. cyanescens* (Miettinen) B.K. Cui & Shun Liu and *C. caesiosimulans* (G.F. Atk.) B.K. Cui & Shun Liu in phylogenetic analyses. Morphologically, *C. cyanescens* and *C. tianshanensis* both have initially white-to-cream-colored upper surfaces with slight bluish-greyish hues, and lack pubescence, but *C. cyanescens* has larger pores (5–6 per mm) and narrower basidiospores (4.7–6.1 × 1.1–1.6 µm, Q = 3.92, [40]). *Cyanosporus tianshanensis* and *C. caesiosimulans* both have white-to-cream-colored upper surfaces; however, *C. caesiosimulans* differs with thicker basidiocarps (context 1–3 mm thick, tubes 1–3 mm long), larger pores (5–7 per mm), and narrower basidiospores (4.2–5.5 × 1.1–1.4 µm, Q = 3.93, [40]).

*Osteina altaiensis* was involved in *Osteina* with strong support (84% ML, 0.99%, MP, 1.00 BPPs) in the phylogenetic analyses. *Osteina altaiensis* and *O. obducta* are phylogenetically closely related. Morphologically, *O. altaiensis* and *O. obducta* have larger basidiocarps with similar pore sizes, but *O. obducta* has a white pileal surface, longer tubes (3 mm long), and wider basidiospores (4–5.2 × 2–2.4 µm, Q = 2.06–2.2, [5]). *Osteina rhodophila* (Spirin & Zmitr.) Bernicchia & Gorjón and *O. altaiensis* both have a distinct brownish hue on the pileal surface and similar pore and basidiospore; however, *O. rhodophila* has smaller and thinner basidiocarps (0.5–3 × 0.1–0.3 cm) and narrower contextual hyphae (2–3 μm, [52]). *Osteina subundosa* (Y.L. Wei & Y.C. Dai) B.K. Cui, Shun Liu & L.L. Shen and *O. altaiensis* both have a distinct brownish hue on the pileal surface and similar basidiospore shapes and sizes, but *O. subundosa* has smaller basidiocarps (2.5–3 × 0.5 cm), larger pores (1–3 per mm), and narrower contextual hyphae (3–6 μm, [53]). *Osteina undosa* and *O. altaiensis* show significant morphological differences; *O. undosa* has effused–reflexed-to-resupinate basidiomata, a white-to-pale buff pileal surface, larger pores (2–3 per mm), and narrower basidiospores (4.5–6 × 1–1.5 μm, Q = 4.22–4.38, [13]).

At present, a total of 17 genera and 102 species of Postiaceae have been reported, including 14 general and 72 species found from China. The species of the Postiaceae predominantly parasitize on gymnosperms, with a minority also capable of growing on angiosperms or on both gymnosperms and angiosperms [7,10]. The gymnosperms mainly include the genera *Pinus*, *Picea*, and *Abies*, while the angiosperms primarily consist of the genera *Quercus*, *Populus*, and *Betula*. Consequently, the type of vegetation has a certain influence on the distribution of the Postiaceae. Geographically, Postiaceae is a family with a north temperate distribution, predominantly found in the alpine and plateau climate zones, temperate monsoon climate zones, and subtropical monsoon climate zones in China. They are less commonly found in temperate continental climate zones and tropical monsoon climate zones [7,10]. The Xinjiang region of China, which has a temperate continental climate, is characterized by a significant proportion of gymnosperms. Until now, 10 species belonging to four genera of Postiaceae, *Amaropostia stiptica*, *A. altaiensis*, *A. tianshanensis*, *Cyanosporus caesiosimulans*, *C. caesius*, *C. cyanescens*, *C. latisporus*, *C. tianshanensis*, *Fuscopostia fragilis* (Fr.) B.K. Cui, L.L. Shen & Y.C. Dai, and *Osteina altaiensis*, were recorded from Xinjiang. As investigations of brown rot fungi in Xinjiang continue to deepen, it is expected that an increasing number of new species will be discovered.

## Figures and Tables

**Table 1:** An inventory of species, specimen information, and their respective GenBank accession numbers for the sequences analyzed phylogenetically in this study.

Species	Sample No.	Locality	GenBank Accessions							References
			ITS	nLSU	mtSSU	nuSSU	RPB1	RPB2	TEF1	
***Amaropostia altaiensis***	**Cui 18983**	**Xinjiang, China**	**PP917921**	**PP917955**	**PP917972**	**PP917938**	**–**	**–**	**PP944601**	**Present study**
***A. altaiensis***	**Cui 19000 (holotype)**	**Xinjiang, China**	**PP917922**	**PP917956**	**PP917973**	**PP917939**	**–**	**PP918933**	**PP944589**	**Present study**
*A. hainanensis*	Cui 13739 (holotype)	Hainan, China	KX900909	KX900979	KX901051	KX901123	KX901171	KX901223	–	[13]
*A. hainanensis*	Cui 5367	Hainan, China	KX900910	KX900980	KX901052	KX901124	KX901172	KX901224	–	[13]
*A. hainanensis*	Dai 15208	Hainan, China	KX900911	KX900981	KX901053	KX901125	–	KX901225	–	[13]
*A. stiptica*	Cui 10043	Jilin, China	KX900906	KX900976	KX901046	KX901119	KX901167	KX901219	–	[13]
*A. stiptica*	Cui 10981	Shandong, China	KX900907	KX900977	KX901047	KX901120	KX901168	KX901220	–	[13]
*A. stiptica*	Cui 18013	Yunnan, China	OM039270	OM039170	OM039205	OM039236	OM037742	OM037768	OM037792	[7]
*A. stiptica*	Cui 17037	Yunnan, China	OK045504	OK045510	OK045498	OK045492	OK076902	OK076930	OK076958	[7]
*A. stiptica*	Cui 9268	Xizang, China	KF727431	KX900978	KX901048	–	–	–	–	[12]
***A. tianshanensis***	**Cui 22197**	**Xinjiang, China**	**PP917919**	**PP917953**	**PP917970**	**PP917936**	**–**	**–**	**PP944599**	**Present study**
***A. tianshanensis***	**Cui 22201 (holotype)**	**Xinjiang, China**	**PP917920**	**PP917954**	**PP917971**	**PP917937**	**–**	**–**	**PP944600**	**Present study**
*Amylocystis lapponica*	FP-105131	Colorado, United States	KY948805	KY948879	–	–	KY948973	–	–	[38]
*A. lapponica*	HHB 13400	Alaska, United States	KC585237	KC585059	AF518667	AF518570	–	–	–	[38]
*A. lapponica*	OKM 4118	Montana, United States	KC585238	KC585060	–	–	–	–	–	[38]
*Antrodia serpens*	Dai 7465	Luxembourg	KR605813	KR605752	KR606013	KR605913	ON424666	KR610832	KR610742	[7]
*Aurantipostia macrospora*	Cui 16604 (holotype)	Tasmania, Australia	MW377258	MW377339	–	MW377417	MW337157	MW337026	MW337089	[7]
*A. macrospora*	Cui 16634	Tasmania, Australia	MW377259	MW377340	–	MW377418	MW337158	MW337027	MW337090	[7]
*A macrospora*	Cui 16671	Tasmania, Australia	MW377260	MW377341	–	MW377419	MW337159	MW337028	MW337091	[7]
*Austropostia brunnea*	Dai 18591A	Victoria, Australia	MW377272	MW377352	–	MW377430	MW337169	MW337038	MW337101	[7]
*A. brunnea*	NLB 1135	Australia	MT536995	MT524530	–	–	–	–	–	Unpublished
*A. hirsuta*	Cui 16660 (holotype)	Tasmania, Australia	MW377267	MW377347	MW382055	MW377425	MW337164	MW337033	MW337096	[7]
*A. hirsuta*	Cui 16661	Tasmania, Australia	MW377268	MW377348	MW382056	MW377426	MW337165	MW337034	MW337097	[7]
*A. hirsuta*	Cui 16662	Tasmania, Australia	MW377269	MW377349	MW382057	MW377427	MW337166	MW337035	MW337098	[7]
*A. pelliculosa*	MR 10592	Chubut, Argentina	JX090102	JX090124	–	–	–	–	–	[39]
*A. pelliculosa*	MR 10671	Neuquén, Argentina	JX090101	JX090123	–	–	–	–	–	[39]
*A. plumbea*	Cui 16550 (holotype)	Victoria, Australia	MW377270	MW377350	MW382058	MW377428	MW337167	MW337036	MW337099	[7]
*A. plumbea*	Cui 16639	Tasmania, Australia	MW377271	MW377351	MW382059	MW377429	MW337168	MW337037	MW337100	[7]
*A. punctata*	MR 11100	Neuquén, Argentina	JX090112	JX090128	–	–	–	–	–	[7]
*A. punctata*	MR 12398	Región X, Chile	JX090111	JX090127	–	–	–	–	–	[7]
*A. subpunctata*	Cui 16675 (holotype)	Tasmania, Australia	MW377273	MW377353	MW382060	MW377431	MW337170	MW337039	MW337102	[7]
*A. subpunctata*	Cui 16685	Tasmania, Australia	MW377274	MW377354	MW382061	MW377432	MW337171	MW337040	MW337103	[7]
*A. subpunctata*	Cui 16686	Tasmania, Australia	MW377275	MW377355	MW382062	MW377433	MW337172	MW337041	MW337104	[7]
*Calcipostia guttulata*	Cui 10018	Jilin, China	KF727432	KJ684978	KX901065	KX901138	KX901181	KX901236	KX901276	[13]
*C. guttulata*	Cui 10028	Jilin, China	KF727433	KJ684979	KX901066	KX901139	KX901182	KX901237	KX901277	[13]
*C. guttulata*	Cui 16274	Yunnan, China	OM039274	OM039174	OM039209	OM039240	OM037746	OM037772	OM037796	[7]
*C. guttulata*	Cui 16281	Yunnan, China	OM039275	OM039175	OM039210	OM039241	OM037747	OM037773	OM037797	[7]
*Cyanosporus alni*	Cui 7185	Hebei, China	KX900879	KX900949	KX901017	KX901092	KX901155	KX901202	KX901254	[13]
*C. alni*	Dai 14845	Poland	KX900880	KX900950	KX901018	KX901093	KX901156	KX901203	KX901255	[13]
*C. alni*	H 7019137	Bratislava, Slovakia	MG137026	–	–	–	–	–	–	[40]
*C. arbuti*	Spirin 8327 (holotype)	Washington, United States	MG137039	–	–	–	–	–	MG137132	[40]
*C. auricomus*	Cui 13518	Inner Mongolia, China	KX900887	KX900957	KX901025	KX901100	–	KX901209	–	[13]
*C. auricomus*	Cui 13519	Inner Mongolia, China	KX900888	KX900958	KX901026	KX901101	–	–	–	[13]
*C. auricomus*	Niemela 8310 (holotype)	Pohjois-Savo, Finland	MG137040	–	–	–	–	–	–	[40]
*C. bifarius*	Cui 16277	Yunnan, China	OL423599	OL423609	OL437196	OL423621	OL444986	OL447000	OL444995	[11]
*C. bifarius*	Cui 17534	Sichuan, China	OL423598	OL423608	OL437195	OL423620	OL444985	OL446999	OL444994	[11]
*C. bifarius*	Spirin 6402 (holotype)	Primorie, Russia	MG137043	–	–	–	–	–	MG137133	[40]
*C. bubalinus*	Cui 16976	Yunnan, China	MW182172	MW182225	MW182208	MW182189	MW191547	MW191563	MW191530	[9]
*C. bubalinus*	Cui 16985 (holotype)	Yunnan, China	MW182173	MW182226	MW182209	MW182190	MW191548	MW191564	MW191531	[9]
*C. caesiosimulans*	Miettinen 16976	New York, United States	MG137054	–	–	–	–	–	MG137137	[40]
*C. caesiosimulans*	Spirin 4199	Khabarovsk, Russia	MG137061	–	–	–	–	–	MG137140	[40]
***C. caesiosimulans***	**Cui 22260**	**Xinjiang, China**	**PP917932**	**PP917966**	**PP917983**	**PP917949**	**–**	**–**	**–**	**Present study**
*C. caesius*	Cui 18630	Nancy, France	OL423600	OL423610	OL437197	OL423622	–	–	OL444996	[11]
*C. caesius*	Schuster 51	Niedersachsen, Germany	MG137045	–	–	–	–	–	–	[40]
*C. caesius*	Miettinen 14156	Uusimaa, Finland	MG137048	–	–	–	–	–	MG137134	[40]
***C. caesius***	**Cui 20518**	**Xinjiang, China**	**PP917933**	**PP917967**	**PP917984**	**PP917950**	**–**	**–**	**–**	**Present study**
*C. caesius* aff GB	K 32425	United Kingdom	AY599575	–	–	–	–	–	–	[40]
*C. caesius* aff GB	K 32713	United Kingdom	AY599576	–	–	–	–	–	–	[40]
*C. coeruleivirens*	Dai 19220	Hunan, China	MW182174	MW182227	MW182210	MW182191	MW191549		MW191532	[9]
*C. coeruleivirens*	Miettinen 12214	Bali, Indonesia	MG137063	–	–	–	–	–	–	[40]
*C. comatus*	Miettinen 14755 (holotype)	Massachusetts, United States	MG137066	–	–	–	–	–	–	[40]
*C. cyanescens*	Miettinen 13602 (holotype)	Uusimaa, Finland	MG137067	–	–	–	–	–	MG137142	[40]
*C. cyanescens*	Miettinen 15919	Huesca, Spain	MG137071	–	–	–	–	–	MG137144	[40]
***C. cyanescens***	**Cui 22122**	**Xinjiang, China**	**PP917934**	**PP917968**	**PP917985**	**PP917951**	**–**	**–**	**PP944597**	**Present study**
***C. cyanescens***	**Cui 22130**	**Xinjiang, China**	**PP917935**	**PP917969**	**PP917986**	**PP917952**	**–**	**–**	**PP944598**	**Present study**
*C. flavus*	Cui 18547 (holotype)	Sichuan, China	MW448564	MW448561	–	MW448557	MW452596	MW452599	MW452601	[11]
*C. flavus*	Cui 18562	Sichuan, China	MW448565	MW448562	–	MW448558	MW452597	MW452600	MW452602	[11]
*C. fusiformis*	Cui 10775	Sichuan, China	KX900868	KX900938	KX901006	KX901081	–	KX901191	KX901245	[13]
*C. fusiformis*	Dai 15036 (holotype)	Guizhou, China	KX900867	KX900937	KX901005	KX901080	–	KX901190	KX901244	[13]
*C. glaucus*	Spirin 5317 (holotype)	Khabarovsk, Russia	MG137078	–	–	–	–	–	–	[40]
*C. glaucus*	Spirin 6580	Khabarovsk, Russia	MG137081	–	–	–	–	–	MG137145	[40]
*C. gossypinus*	LY BR 6658	Vaucluse, France	–	–	–	–	–	–	MG137146	[40]
*C. hirsutus*	Cui 17083 (holotype)	Yunnan, China	MW182179	MW182233	MW182214	MW182197	MW191554	MW191568	MW191538	[9]
*C. hirsutus*	Cui 17342	Sichuan, China	OL423602	OL423612	OL437199	OL423624	OL444988	OL447002	OL444998	[11]
*C. hirsutus*	Cui 17343	Sichuan, China	OL423601	OL423611	OL437198	OL423623	OL444987	OL447001	OL444997	[11]
***C. latisporus***	**Cui 16827 (holotype)**	**Xinjiang, China**	**PP917925**	**PP917959**	**PP917976**	**PP917942**	**PP918931**	**PP918932**	**PP944588**	**Present study**
*C. livens*	Miettinen 17177 (holotype)	New York, United States	MG137082	–	–	–	–	–	MG137147	[40]
*C. livens*	Spirin 8728	Washington, United States	MG137090	–	–	–	–	–	MG137150	[40]
*C. luteocaesia*	LY BR 2605	France	MG137091	–	–	–	–	–	–	[40]
*C. magnus*	Cui 16983	Yunnan, China	MW182180	MW182234	MW182215	MW182198	MW191555	MW191569	MW191539	[9]
*C. magnus*	Dai 21105	Chongqin, China	OL423603	OL423613	OL437200	OL423625	OL444989	OL447003	OL444999	[11]
*C. magnus*	Miettinen 10634 (holotype)	Jilin, China	KC595944	KC595944	–	–	–	–	MG137151	[40]
*C. mediterraneocaesius*	LY BR 4274	Bonnieux, France	KX900886	–	KX901024	KX901099	–	–	–	[13]
*C. microporus*	Cui 11014 (holotype)	Yunnan, China	KX900878	KX900948	KX901016	KX901091	–	KX901201	–	[13]
*C. microporus*	Dai 11717	Yunnan, China	KX900877	KX900947	KX901015	KX901090	–	KX901200	–	[13]
*C. nothofagicola*	Cui 16697 (holotype)	Tasmania, Australia	MW182181	MW182235	MW182216	MW182199	MW191556	MW191570	MW191540	[9]
*C. nothofagicola*	Dai 18765	Tasmania, Australia	MW182182	MW182236	MW182217	MW182200	MW191557		MW191541	[9]
*C. piceicola*	Cui 10626 (holotype)	Sichuan, China	KX900862	KX900932	KX901001	KX901075		KX901185		[13]
*C. piceicola*	Cui 12158	Xizang, China	KX900866	KX900936	KX901004	KX901079	KX901153	KX901189	KX901243	[13]
*C. populi*	Cui 17087a	Yunnan, China	MW182183	MW182237	MW182218	MW182201	MW191558	MW191571	MW191542	[9]
*C. populi*	Cui 17557	Sichuan, China	OL423605	OL423615	OL437202	OL423627	OL444991	OL447005	OL445001	[11]
*C. populi*	Dai 18934	Qinghai, China	OL423604	OL423614	OL437201	OL423626	OL444990	OL447004	OL445000	[11]
*C. populi*	Miettinen 17043 (holotype)	NewYork, United States	MG137092	–	–	–	–	–	MG137153	[40]
*C. rigidus*	Cui 17032 (holotype)	Yunnan, China	OL423606	OL423617	OL437204	OL423629	OL444993	–	OL445003	[11]
*C. simulans*	Miettinen 20422	Satakunta, Finland	MG137110	–	–	–	–	–	MG137160	[40]
*C. simulans*	Niemela 8846	Satakunta, Finland	MG137103	–	–	–	–	–	–	[40]
*C. subcaesius*	H 7034976	Isère, France	MG137116	–	–	–	–	–	–	[40]
*C. subcaesius*	JV 0110/24	Jihomoravský, Czech Republic	MG137117	–	–	–	–		MG137164	[40]
*C. subhirsutus*	Cui 11330	Fujian, China	KX900873	KX900943	KX901011	KX901086	–	KX901196	KX901250	[13]
*C. subhirsutus*	Dai 14892 (holotype)	Guizhou, China	KX900871	KX900941	KX901009	KX901084	–	KX901194	KX901248	[13]
***C. tianshanensis***	**Cui 22109**	**Xinjiang, China**	**PP917923**	**PP917957**	**PP917974**	**PP917940**	**–**	**–**	**PP944596**	**Present study**
***C. tianshanensis***	**Cui 22709 (holotype)**	**Xinjiang, China**	**PP917924**	**PP917958**	**PP917975**	**PP917941**	**–**	**–**	**–**	**Present study**
*C. submicroporus*	Cui 16306	Yunnan, China	MW182184	MW182239	MW182220	MW182203	MW191560	MW191573	MW191544	[9]
*C. submicroporus*	Cui 18156 (holotype)	Yunnan, China	MW182186	MW182241	MW182222	MW182205	–	MW191574	–	[9]
*C. subungulatus*	Cui 18046 (holotype)	Yunnan, China	MW448566	MW448563	MW448560	MW448559	MW452598	–	MW452603	[11]
*C. subungulatus*	Zhao 10833	Yunnan, China	MW742586	OL423616	OL437203	OL423628	OL444992	–	OL445002	[11]
*C. subviridis*	Penttila 14376	Pohjois-Karjala, Finland	–	–	–	–	–	–	MG137165	[40]
*C. subviridis*	Spirin 8774a	Washington, United States	MG137120	–	–	–	–	–	MG137166	[40]
*C. tenuicontextus*	Cui 16280 (holotype)	Yunnan, China	OL423607	OL423618	OL437205	OL423630	–	–	OL445004	[11]
*C. tenuicontextus*	Zhao 813	Yunnan, China	MG231802	OL423619	OL437206	OL423631	–	–	OL445005	[11]
*C. tenuis*	Cui 10788 (holotype)	Sichuan, China	KX900885	KX900955	KX901023	KX901098	KX901161	KX901208	–	[13]
*C. tenuis*	Dai 12974	Sichuan, China	KX900884	KX900954	KX901022	KX901097	KX901160	KX901207	KX901258	[13]
*C. tricolor*	Cui 10790	Sichuan, China	KX900875	KX900945	KX901013	KX901088	–	KX901198	KX901252	[13]
*C. tricolor*	Cui 12233 (holotype)	Xizang, China	KX900876	KX900946	KX901014	KX901089	–	KX901199	KX901253	[13]
*C. ungulatus*	Cui 10778	Sichuan, China	KX900870	KX900940	KX901008	KX901083	–	KX901193	KX901247	[13]
*C. ungulatus*	Dai 12897 (holotype)	Sichuan, China	KX900869	KX900939	KX901007	KX901082	KX901154	KX901192	KX901246	[13]
*C. yanae*	HK 27454 (holotype)	Sakha, Russia	MG137121	–	–	–	–	–	MG137167	[40]
*C. yanae*	HK 27606	Sakha, Russia	MG137122	–	–	–	–	–	MG137168	[40]
*Cystidiopostia hibernica*	Cui 17624	Sichuan, China	MW377277	MW377357	MW382064	MW377435	MW337173	–	MW337105	[7]
*C. hibernica*	Cui 2658	Zhejiang, China	KX900905	KX900975	KX901045	KX901118	–	KX901218	–	[13]
*C. inocybe*	LY BR 3703	France	KX900903	KX900973	KX901044	KX901116	–	–	KX901267	[13]
*C. pileata*	Cui 10034	Jilin, China	KX900908	KX900956	KX901050	KX901122	KX901170	KX901222	KX901269	[13]
*C. pileata*	Cui 5721	Liaoning, China	KF699127	KX900960	KX901049	KX901121	KX901169	KX901221	KX901268	[13]
*C. subhibernica*	Cui 17095 (holotype)	Yunnan, China	MW377278	MW377358	MW382065	MW377436	MW337174	MW337042	MW337106	[7]
*C. subhibernica*	Dai 17621	Sichuan, China	OM039276	OM039176	OM039211	OM039242	OM037749	OM037774	OM037798	[7]
*Fomitopsis betulina*	Cui 17121	Yunnan, China	OL621853	OL621242	OL621753	OL621779	ON424683	OL588969	OL588982	[11]
*Fuscopostia duplicata*	Cui 10366	Yunnan, China	KF699124	KJ684975	KR606026	KR605927	KX901173	KR610844	KR610755	[6]
*F. duplicata*	Dai 13411 (holotype)	Zhejiang, China	KF699125	KJ684976	KR606027	KR605928	KX901174	KR610845	KR610756	[6]
*F. fragilis*	Cui 10020	Jilin, China	KX900912	KX900982	KX901054	KX901126	ON424693	KX901226	KX901270	[13]
*F. fragilis*	Cui 10088	Jilin, China	KF699120	KJ684977	KT893749	KX901127	ON424692	KT893745	KT893747	[6]
*F. lateritia*	Dai 2652	Helsinki, Finland	KX900913	KX900983	–	–	–	–	–	[13]
*F. lateritia*	KUO 0211531	Khabarovsk, Russia	JF950567	–	–	–	–	–	–	[41]
*F. leucomallella*	Cui 9577	Xizang, China	KF699122	KJ684982	KX901055	KX901128	KX901175	KX901227	KX901271	[13]
*F. leucomallella*	Cui 9599	Xizang, China	KF699123	KJ684983	KX901056	KX901129	KX901176	KX901228	KX901272	[13]
*F. subfragilis*	Cui 16282	Yunnan, China	MW377296	MW377375	MW382082	MW377454	MW337189	MW337057	MW337123	[7]
*F. subfragilis*	Cui 16302 (holotype)	Yunnan, China	MW377297	MW377376	MW382083	MW377455	MW337190	MW337058	MW337124	[7]
*Jahnoporus brachiatus*	X 3232 (holotype)	Khabarovsk, Russia	KU165781	–	–	–	–	–	–	[42]
*J. hirtus*	AFTOL ID 1687	Washington, United States	DQ911605	DQ911606	–	DQ911607	–	DQ911608	–	[43]
*J. hirtus*	Spinosa 10X2014	Washington, United States	KU165784	–	–	–	KY949044	–	–	[42]
*J. oreinus*	X 3241 (holotype)	Khabarovsk, Russia	KU165785	–	–	–	–	–	–	[42]
*Nothofagiporus venatus*	Cui 16616	Tasmania, Australia	MW377310	MW377388	MW382091	MW377467	MW337196	MW337067	MW337133	[7]
*N. venatus*	Cui 16617	Tasmania, Australia	MW377311	MW377389	MW382092	MW377468	MW337197	MW337068	MW337134	[7]
*N. venatus*	Cui 16644	Tasmania, Australia	ON417170	ON417220	ON417084	ON417034	–	ON424786	ON424848	[7]
*Oligoporus podocarpi*	Dai 22042 (holotype)	Hainan, China	MW937877	MW937884	MW937891	MW937870	MZ005579	MZ082976	MZ082982	[15]
*O. podocarpi*	Dai 22043	Hainan, China	MW937878	MW937885	MW937892	MW937871	MZ005580	MZ082977	MZ082983	[7]
*O. rennyi*	Cui 17054	Yunnan, China	OK045508	OK045514	OK045502	OK045496	OK076906	OK076934	OK076962	[7]
*O. rennyi*	Dai 21016	Belarus	ON417173	ON417223	ON417085	ON417037	ON424713	ON424789	ON424851	[7]
*O. romellii*	Dai 21034	Belarus	MW377312	MW377390	MW382093	MW377469	MW337198	ON424790	MW337135	[7]
*O. romellii*	Dai 23576	Xizang, China	ON417174	ON417224	ON417086	ON417038	ON424714	ON424791	ON424852	[7]
*O. sericeomollis*	Cui 9560	Xizang, China	KX900919	KX900989	KX901067	KX901140	KX901183	ON424792	ON424853	[13]
*O. sericeomollis*	Dai 23473	Xizang, China	ON417175	ON417225	ON417087	ON417039	ON424715	ON424793	ON424854	[7]
*Osteina obducta*	Cui 10074	Jilin, China	KX900924	KX900994	KX901071	KX901144	–	KX901240	–	[13]
*O. obducta*	Cui 9832	Heilongjiang, China	KX900925	KX900995	–	–	–	–	–	[13]
*O. obducta*	Cui 9959	Jilin, China	KX900923	KX900993	KX901070	KX901143	–	KX901239	–	[13]
*O. undosa*	Cui 16651	Tasmania, Australia	MW377313	MW377391	MW382094	MW377470	MW337199	MW337069	MW337136	[7]
*O. undosa*	Dai 6942	Jilin, China	KX900922	KX900992	–	–	–	–	–	[13]
*O. undosa*	Dai 7105	Jilin, China	KX900921	KX900991	KX901069	KX901142	–	KX901238	–	[13]
*O. undosa*	L 10830	North Carolina, United States	KC585396	KC585229	–	–	–	–	–	[38]
*O. undosa*	L 6646	Colorado, United States	KC585399	KC585232	–	–	–	–	–	[38]
***O. altaiensis***	**Cui 20555**	**Xinjiang, China**	**PP917926**	**PP917960**	**PP917977**	**PP917943**	**–**	**–**	**PP944590**	**Present study**
***O. altaiensis***	**Cui 20920 (holotype)**	**Xinjiang, China**	**PP917927**	**PP917961**	**PP917978**	**PP917944**	**–**	**–**	**PP944591**	**Present study**
***O. altaiensis***	**Cui 20963**	**Xinjiang, China**	**PP917928**	**PP917962**	**PP917979**	**PP917945**	**–**	**–**	**PP944592**	**Present study**
***O. altaiensis***	**Cui 20964**	**Xinjiang, China**	**PP917929**	**PP917963**	**PP917980**	**PP917946**	**–**	**–**	**PP944593**	**Present study**
***O. altaiensis***	**Cui 20970**	**Xinjiang, China**	**PP917930**	**PP917964**	**PP917981**	**PP917947**	**–**	**–**	**PP944594**	**Present study**
***O. altaiensis***	**Cui 20972**	**Xinjiang, China**	**PP917931**	**PP917965**	**PP917982**	**PP917948**	**–**	**–**	**PP944595**	**Present study**
*Postia amurensis*	Cui 1044	Liaoning, China	KX900902	KX900972	KX901043	–	–	–	–	[13]
*P. amurensis*	Dai 903 (holotype)	Jilin, China	KX900901	KX900971	KX901042	–	–	–	–	[13]
*P. crassicontexta*	Cui 16637 (holotype)	Tasmania, Australia	MW377315	MW377393	MW382096	MW377472	MW337200	MW337071	MW337138	[7]
*P. cylindrica*	Dai 17941	Hubei, China	ON417183	ON417233	ON417091	ON417047	–	–	ON424862	[7]
*P. cylindrica*	Dai 23087	Yunnan, China	ON417182	ON417232	ON417090	ON417046	–	–	ON424861	[7]
*P. hirsuta*	Cui 11237 (holotype)	Shanxi, China	KJ684970	KJ684984	KX901038	KX901113	–	–	KX901266	[13]
*P. hirsuta*	Cui 18347	Hunan, China	OM039286	OM039186	OM039221	OM039253	–	ON424800	OM037809	[7]
*P. lactea*	Cui 17334	Sichuan, China	OM039287	OM039187	OM039222	OM039254	OM037753	OM037782	OM037810	[7]
*P. lactea*	Cui 17790	Sichuan, China	OM039288	OM039188	OM039223	OM039255	OM037754	OM037783	OM037811	[7]
*P. lowei*	Cui 18366	Sichuan, China	OM039289	OM039189	OM039224	OM039256	–	ON424801	ON424863	[7]
*P. lowei*	Cui 9585	Xizang, China	KX900898	KX900968	KX901035	KX901110	–	–	–	[13]
*P. ochraceoalba*	Cui 17044	Yunnan, China	OM039290	OM039190	OM039225	OM039257	OM037755	OM037784	OM037812	[7]
*P. ochraceoalba*	Cui 17047	Yunnan, China	OM039291	OM039191	OM039226	OM039258	OM037756	OM037785	OM037813	[7]
*P. sublowei*	Cui 17460	Sichuan, China	OM039294	OM039194	OM039229	OM039261	OM037759	ON424802	ON424864	[7]
*P. sublowei*	Cui 9352	Xizang, China	KX900899	KX900969	KX901036	KX901111	ON424723	–	KX901264	[7]
*P. tephroleuca*	Cui 17329	Sichuan, China	OK045509	OK045515	OK045503	OK045497	OK076907	OK076935	OK076963	[7]
*P. tephroleuca*	Cui 17560	Sichuan, China	OM039295	OM039195	OM039230	OM039262	OM037760	OM037788	OM037816	[7]
*Ptychogaster albus*	Dai 21035	Belarus	OM039293	OM039193	OM039228	OM039260	OM037758	OM037787	OM037815	[7]
*P. albus*	Dai 23535	Xizang, China	ON417184	ON417235	ON417092	ON417048	ON424724	ON424804	ON424866	[7]
*P. albus*	Dai 23618	Xizang, China	OM039292	OM039192	OM039227	OM039259	OM037757	OM037786	OM037814	[7]
*Spongiporus balsameus*	Cui 9835	Heilongjiang, China	KX900916	KX900986	KX901061	KX901134	–	KX901233	–	[13]
*S. balsameus*	Dai 22714	Yunnan, China	ON417194	ON417246	ON417101	ON417058	–	ON424814	ON424880	[7]
*S. floriformis*	Cui 10292	Yunnan, China	KM107899	KM107904	KX901058	KX901131	KX901178	KX901230	KX901274	[13]
*S. floriformis*	Cui 17066	Yunnan, China	OM039300	OM039200	OM039231	OM039265	OM037762	ON424815	OM037818	[7]
*S. floriformis*	Dai 13887	Yunnan, China	KX900914	KX900984	KX901057	KX901130	KX901177	KX901229	KX901273	[13]
*S. gloeoporus*	Cui 10401	Yunnan, China	KX900915	KX900985	KX901060	KX901133	ON424742	KX901232	ON424881	[13]
*S. gloeoporus*	Cui 17813	Singapore	OM039301	OM039201	OM039232	OM039266	OM037763	ON424816	OM037819	[7]
*S. zebra*	Cui 9973	Jilin, China	KX900917	KX900987	KX901062	KX901135	KX901179	KX901234	–	[13]
*S. zebra*	Dai 7131 (holotype)	Jilin, China	KF727430	KM190902	KX901063	KX901136	KX901180	KX901235	–	[13]
*Tenuipostia dissecta*	Cui 16555	Victoria, Australia	MW377330	MW377406	MW382106	MW377487	MW337207	ON424818	MW337149	[7]
*T. dissecta*	Cui 16560	Victoria, Australia	MW377331	MW377407	MW382107	MW377488	MW337208	ON424819	MW337150	[7]
*T. dissecta*	Cui 16653	Tasmania, Australia	OM039302	OM039202	OM039233	OM039267	OM037764	OM037789	OM037820	[7]
*T. dissecta*	Dai 18747	Tasmania, Australia	OM039303	OM039203	OM039234	OM039268	OM037765	OM037790	OM037821	[7]

The newly generated sequences were accentuated in bold.

## Data Availability

The data and results of this study are available upon reasonable request. Please contact the main author of this publication.
